# Supplementary material for: Informatics investigations into anti-thyroid drug induced agranulocytosis associated with multiple HLA-B alleles
Source: PLoS One. 2020 Feb 6;15(2):e0220754. doi: 10.1371/journal.pone.0220754 (PMC7004376; doi:10.1371/journal.pone.0220754)
Supplement: S1 File — Supporting figures and tables. (DOCX) [file pone.0220754.s001.docx]

**Supporting Information**

**i. Control allele selection**

Case and control frequencies were obtained from the association studies for Hallberg *et al.* [1] and Chen *et al.* [2]. No control frequency data was available for the He *et al.* study [3]. The top ten most common allele frequencies from AFND [4] were obtained for Han Northern China and the European populations used in the Hallberg *et al*. study: Sweden, France, Spain and Germany. These were then compared with the case and control frequencies obtained from the association studies, for alleles with healthy control frequency over 3%. Our control alleles were selected as alleles where the study control allele frequency or healthy individual frequencies sourced from AFND were similar to or greater than the cases.

Of the control alleles selected from the He *et al.* study, five were shown to have frequencies greater in controls than cases (S1 Fig c-d). B*15:01, B*40:06, B *46:01, B*51:01 and B*54:01. These alleles generally showed similar frequencies to those obtained from AFND [4] for healthy Han Northern China individuals and so can be assumed to be accurate. B*46:01 and B*51:01 showed lower frequencies in the AFND Northern Han population than both the case and control. This may mean that these control frequencies were not representative of the larger population and so may hide a potential association. However, AFND frequencies for Chinese populations, including other Han populations, showed frequencies similar to the control groups of the study (data not shown) and so it is likely the control was indeed representative of the larger population. As the population sample of the AFND Han Northern population is unknown, comparisons are best made between the case risk and control populations, where available. As a result, these alleles were included as selected controls.

The Hallberg *et al*. study reports allele data for only the top ten markers, showing the associated alleles with highest significance, and so less data is available for the Caucasian populations. AFND frequencies were compared to case and control frequencies, where available (S1 Fig a-b). Of the fifteen alleles with AFND frequency greater than 3% for one or more of the European populations, B*15:01, was seen to have control frequencies greater than the risk group. This allele, along with one other, B*51:01, was also selected as a control from the Han Northern China group. Although no case or control data from the Hallberg *et al.* study was available, this was a common allele found in Caucasian populations and is therefore likely not associated in Caucasian or Han Chinese populations.


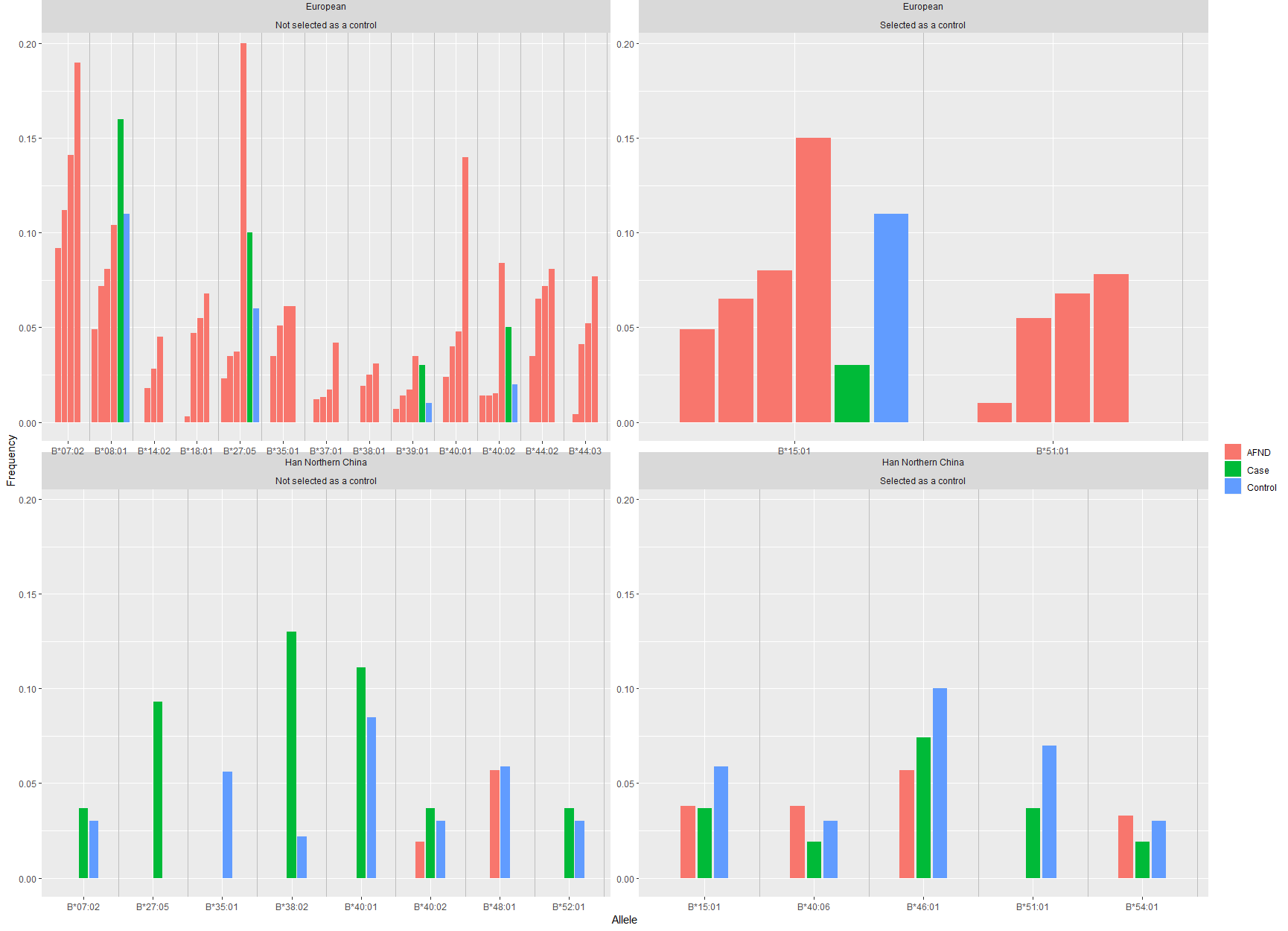


d)

c)

b)

a)

***Figure A: Bar chart of allele frequencies for investigated populations.*** *Healthy population frequencies obtained from AFND [4] along with Case and Control frequencies for alleles from the He et al. study [3] (Han Northern China) with control frequency over 3% and alleles from the Hallberg et al. study [1] with AFND frequency greater than 3% in at least one population (Sweden, France, Spain, or Germany). Panels show alleles separated not only by population but also separating those alleles that were selected as suitable controls.*

**ii. Docking analysis**

The docking process for methimazole and propylthiouracil was repeated, expanding the analysis to give 100 predicted poses for each of the drug-allele combinations. In order to assign the binding position of the predicted poses in each case, poses were plotted based on the 3D PDBQT output co-ordinates and k-mean cluster analysis was used to determine the most favourable binding pocket for each predicted pose. This cluster analysis was able to correctly determine the binding position (B or F pocket) for each pose and was used to create boxplots showing the docking scores for each drug-allele combinations, showing the favouring pocket of poses for each combination.


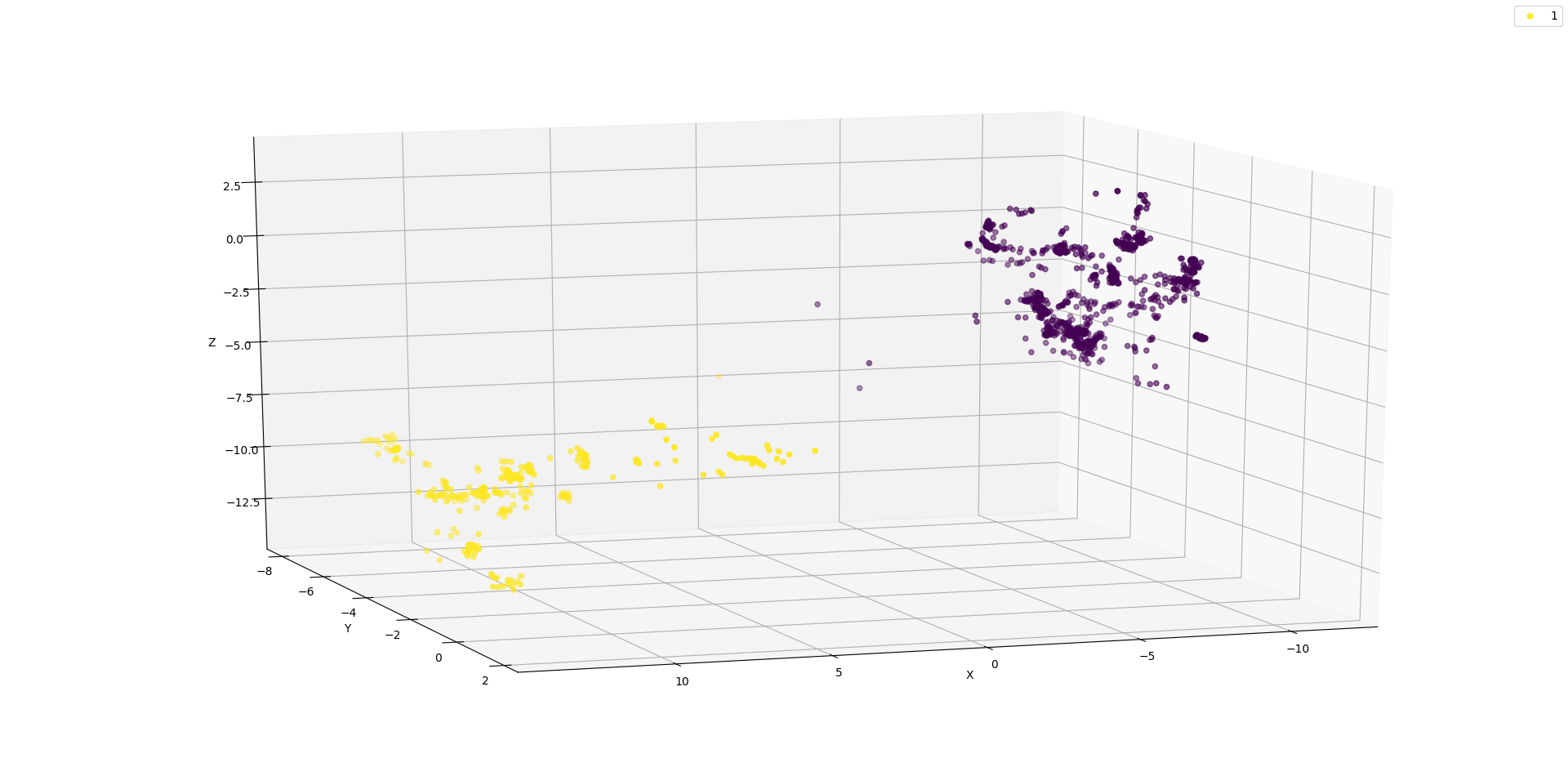

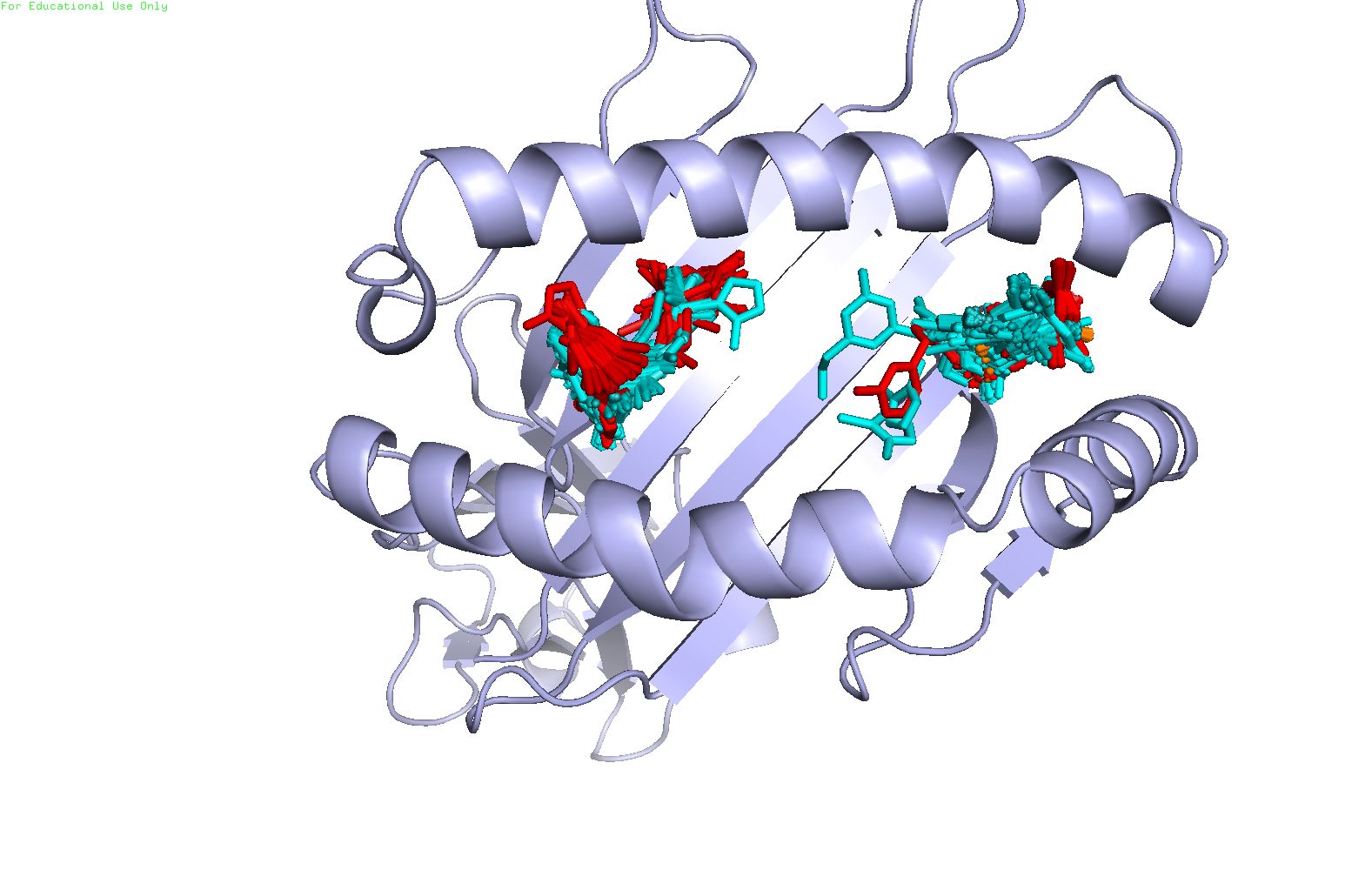


***Figure B: Clustering analysis and docking predicted docking poses of 100 runs.*** *F pocket predictions shown in purple, B pocket predictions shown in yellow – automatically assigned into groups using k-means analysis. Top scoring docking poses of methimazole and propylthiouracil for B*38:02_M and B*27:05_S risk alleles (red), B*38:01_M non-associated allele (orange) and the control alleles (blue) B*15:01_S, B*40:06_M, B*46:01_M, B*51:01_S and B*54:01_M, using peptide binding groove search space for AutoDockFR.*

**iii. Sequence comparison**

Sequence comparison was used to assess how likely it is to find a single residue which is unique to one risk and one other “unrelated” allele, where unrelated is defined as being from a different allele group and different main branch as observed on MHCCluster. For each of the risk alleles (B*27:05 and B*38:02) pairwise comparisons were made at each residue for each of the selected control alleles. B*38:01 was removed from this analysis due to the high sequence similarity to B*38:02 and the uncertainty of association. Counts were made for alleles with matches between the risk and only one control. A probability could then be calculated from the number of alleles that found at least one individual match.

These matches were then compared to the predicted binding positions for each of the alleles to assess the probability of these matches also being close to the binding position and therefore likely involved in the mechanism of binding. Residues contributing to the formation of the B and F pockets, as seen in S4 Fig [5, 6], were considered as potentially interacting residues. This approach allows us to consider the bias of selecting specific residues of interest based on multiple sequence alignments and proximity to predicted binding poses, as we can estimate how likely this event is to occur at random within an unrelated set of alleles.

When searching for matches between each risk allele, B*27:05 and B*38:02, with each of the unrelated controls, it was found that both risk alleles had unique residues i.e. a polymorphism uniquely shared with one other sequence, with three of the five control alleles; B*40:06, B*54:01 and B*51:01. This shows us that finding one or more residues which are unique to the risk and one other sequence is a very common occurrence.

When this method was extended to include the probability of finding these matched alleles alongside the predicted binding positions for the risk drugs, it was found that, comparing to B*27:05, B*40:06 showed 9 unique matches, of which 2 residues are involved in the formation of the B pocket and are therefore potential interaction residues (S3 Fig). Comparing to B*38:02, B*51:01 showed 4 unique matches, of which 2 positions are potential interaction residues within the F pocket (S3 Fig). Table 1 shows the counts for the number of residues unique for each risk-target combination and how many of these are also considered potential interaction residues within the B or F pocket. For B*40:06 and B*54:01, all poses were seen in the F-pocket, whereas for B*51:01, poses were seen in both the B and the F-pockets. The unique residues matching for B*40:06 were therefore not seen forming interactions with the associated drugs (S11-S12 Fig). For B*51:01 interactions were seen with both unique positions (77 and 81) for propylthiouracil but not methimazole (S11-S12 Fig). Therefore, having a uniquely shared amino acid polymorphism between two alleles and near the binding site could occur by chance, even for distantly related alleles.


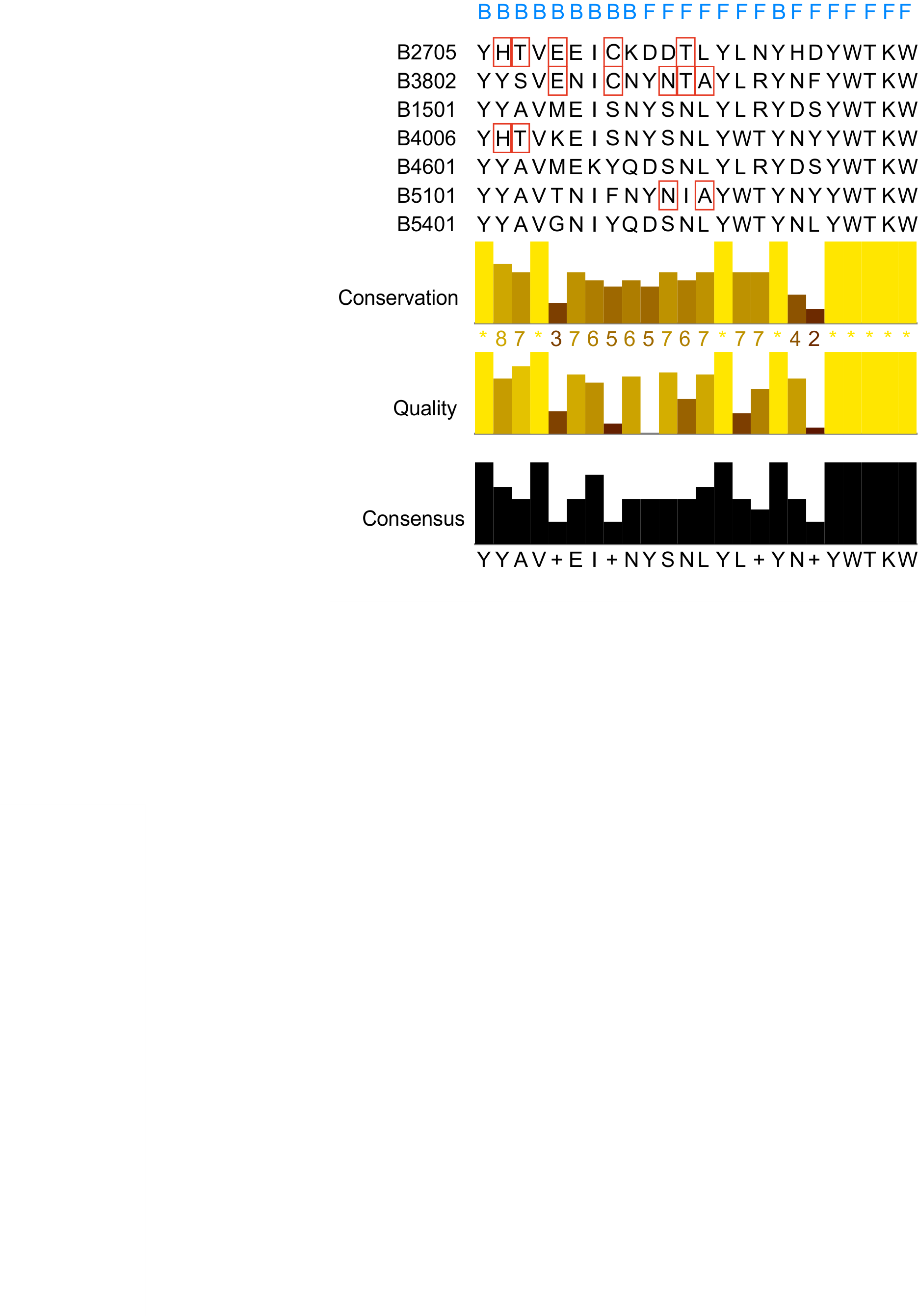


***Figure C: Alignment of positions potentially involved in binding for the B and F pockets.*** *Unique matches identified at these positions are shown highlighted in red.*

***Table A: Unique residues found and residues within 5Å of binding positions.***

| Target allele | Positions found unique to B*27:05 and target allele | Unique positions for B*27:05 also potential interaction positions | Positions found unique to B*38:02 and target allele | Unique positions for B*38:02 also potential interaction positions |
| --- | --- | --- | --- | --- |
| **B*27:05** | **N/A** | **N/A** | **3** | **3** |
| **B*38:02** | **3** | **3** | **N/A** | **N/A** |
| B*40:06 | 9 | 2 | 4 | 0 |
| B*54:01 | 1 | 0 | 1 | 0 |
| B*51:01 | 2 | 0 | 4 | 2 |
| B*15:01 | 0 | 0 | 0 | 0 |
| B*46:01 | 0 | 0 | 0 | 0 |

*Counts of residues positions which were found unique for each risk-target allele combination, the number of the unique allele which were also found lying close to the predicted binding site for each risk-target allele combination. Bold denotes risk alleles, where residues unique and potential interaction positions include Thr80 residue.*

**iv. Tables and figures**

**Table B: Structures obtained for risk and control alleles**

| *Association status* | *Allele* | *Structure* |
| --- | --- | --- |
| *Risk* | *B*27:05_S* | *PDB ID 1OGT [7]* |
|  | *B*38:02_M* | *Homology model using B*14:02 (3BVN [8]), B*39:01 (4O2C [9]) and B*18:01 (4XXC [10]) as templates* |
| *Suspected risk* | *B*38:01_M* | *Homology model using B*53:01 (1A1M [11]), B*44:03 (4JQX [12]) and B*15:01 (5TXS [13]) as templates* |
| *Control* | *B*15:01_S* | *PDB ID 1XR9 [14]* |
|  | *B*40:06_M* | *Homology model using B*41:03 (3LN4 [15]), B*40:02 (5IEK [16]) and B*07:02 (6AT5 [17]) as templates* |
|  | *B*46:01_M* | *Homology model using B*15:01 (1XR9 [14]), B*35:08 (1ZHL [18]) and B*07:02 (6AT5 [17]) as templates* |
|  | *B*51:01_S* | *PDB ID 1E27 [19]* |
|  | *B*54:01_M* | *Homology model using B*35:01 (1A9B [20]), B*14:02 (3BVN [8]) and B*07:02 (6AT5 [17]) as templates* |

**Table C: Summary of molecular docking positions for investigated compounds.**

| Status | Allele | Drug | Lowest score | Poses in B | Median Score | Poses in F | Median Score |
| --- | --- | --- | --- | --- | --- | --- | --- |
| Risk | B*27:05_S | MZY (exp) | F | 0 | 0 | 10 | -3.070614 |
| Risk | B*38:02_M | MZY (exp) | F | 0 | 0 | 10 | -3.9220455 |
| Possible risk | B*38:01_M | MZY (exp) | B | 3 | -3.341496 | 7 | -3.227518 |
| Control | B*15:01_S | MZY (exp) | B | 10 | -3.462575 | 0 | 0 |
| Control | B*40:06_M | MZY (exp) | F | 0 | 0 | 10 | -3.3919205 |
| Control | B*46:01_M | MZY (exp) | B | 9 | -3.643768 | 1 | -3.464709 |
| Control | B*51:01_S | MZY (exp) | F | 1 | -3.071087 | 9 | -3.416686 |
| Control | B*54:01_M | MZY (exp) | F | 0 | 0 | 10 | -3.6175535 |
| Risk | B*27:05_S | TUL (exp) | F | 5 | -3.778168 | 5 | -3.790127 |
| Risk | B*38:02_M | TUL (exp) | F | 0 | 0 | 10 | -4.7819135 |
| Possible risk | B*38:01_M | TUL (exp) | F | 0 | 0 | 10 | -4.291029 |
| Control | B*15:01_S | TUL (exp) | B | 7 | -4.306454 | 3 | -4.287779 |
| Control | B*40:06_M | TUL (exp) | F | 0 | 0 | 10 | -4.2255945 |
| Control | B*46:01_M | TUL (exp) | B | 10 | -4.3200245 | 0 | 0 |
| Control | B*51:01_S | TUL (exp) | B | 2 | -4.416955 | 8 | -4.3621625 |
| Control | B*54:01_M | TUL (exp) | F | 0 | 0 | 10 | -4.1813925 |
| Risk | B*27:05_S | DMI | B | 9 | -3.470353 | 1 | -3.362181 |
| Risk | B*38:02_M | DMI | B | 3 | -3.419452 | 7 | -3.464552 |
| Possible risk | B*38:01_M | DMI | F | 1 | -3.334048 | 9 | -3.491955 |
| Control | B*15:01_S | DMI | B | 9 | -3.563261 | 1 | -3.291783 |
| Control | B*40:06_M | DMI | B | 9 | -3.402275 | 1 | -3.382025 |
| Control | B*46:01_M | DMI | B | 10 | -3.348874 | 0 | 0 |
| Control | B*51:01_S | DMI | B | 9 | -3.371843 | 1 | -3.183533 |
| Control | B*54:01_M | DMI | B | 10 | -3.3084245 | 0 | 0 |
| Risk | B*27:05_S | EV0 | B | 10 | -6.6703375 | 0 | 0 |
| Risk | B*38:02_M | EV0 | F | 4 | -5.811402 | 6 | -6.092657 |
| Possible risk | B*38:01_M | EV0 | F | 0 | 0 | 10 | -5.8363895 |
| Control | B*15:01_S | EV0 | B | 9 | -6.255081 | 1 | -5.895086 |
| Control | B*40:06_M | EV0 | B | 10 | -5.6211235 | 0 | 0 |
| Control | B*46:01_M | EV0 | B | 10 | -6.4380795 | 0 | 0 |
| Control | B*51:01_S | EV0 | B | 9 | -5.932976 | 1 | -5.590286 |
| Control | B*54:01_M | EV0 | F | 0 | 0 | 10 | -5.9992105 |

Position of the lowest scoring poses for each of the drug-allele combinations, along with the number of poses in each pocket and the median of the pose scores in each pocket for each of the alleles using a search space covering the peptide binding groove. Scores given as kcal/mol.


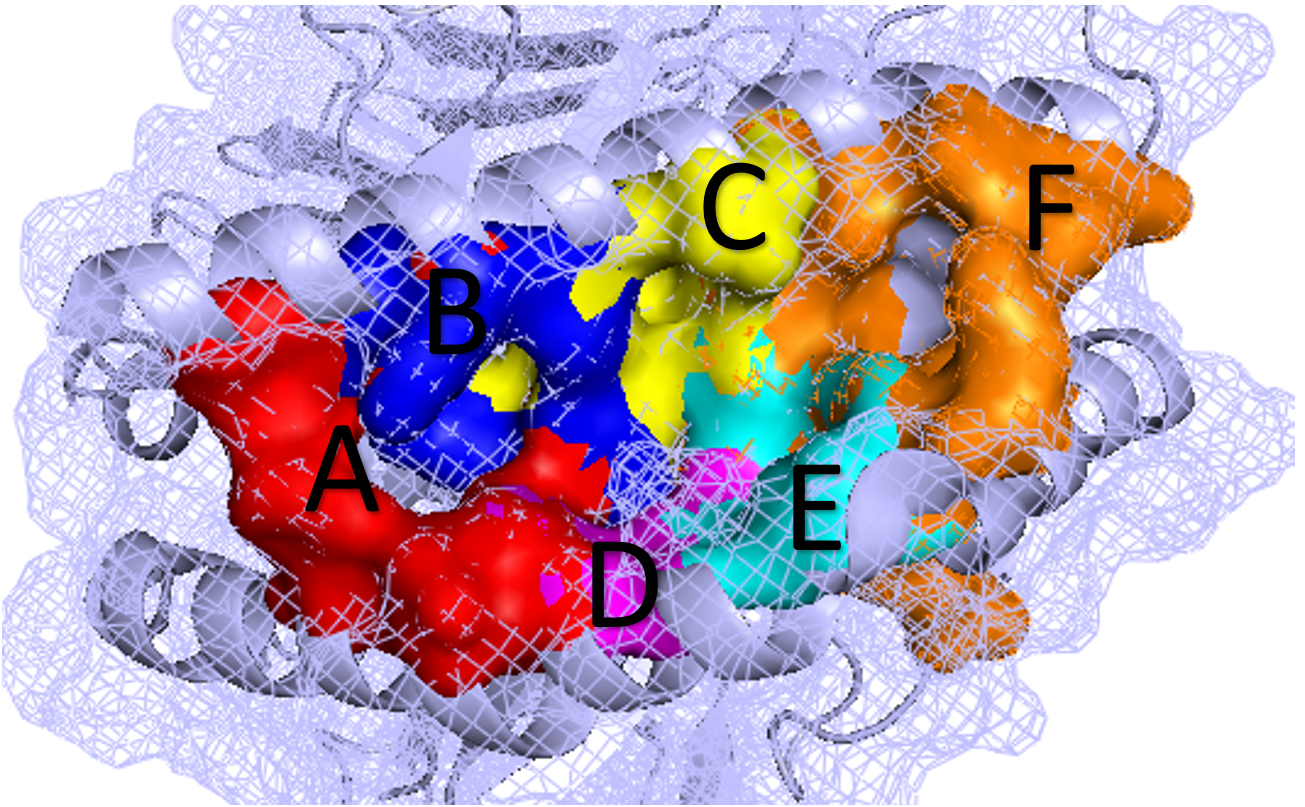


***Figure D: Organisation of subsites along the HLA peptide binding groove.*** *The six subsites along the peptide biding groove (A-F) are shown highlighted [5, 6]. Image created using PyMOL [21].*

*
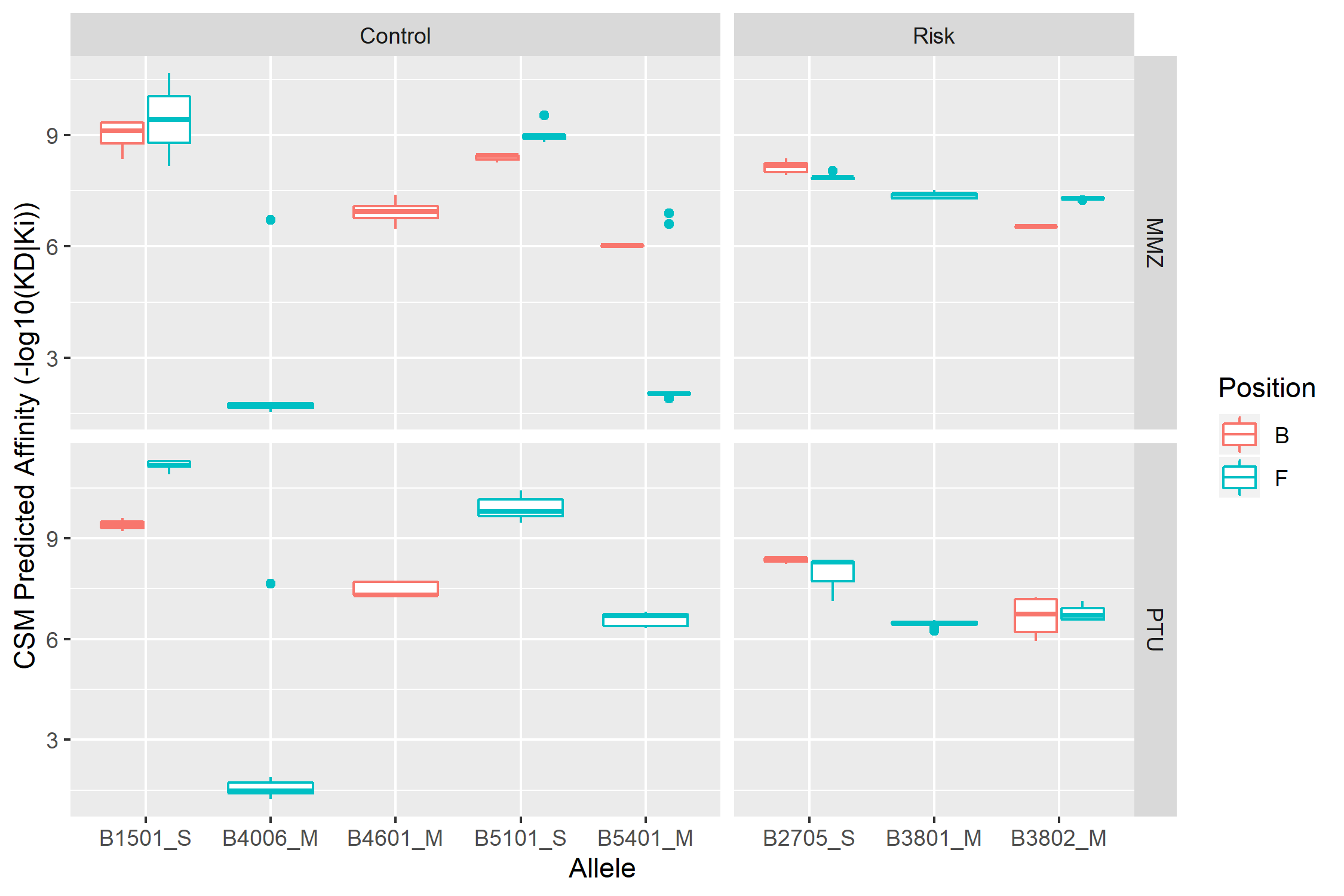
*

***Figure E:* CSM-lig predicted affinity.** Boxplots showing CSM-lig predicted affinity of poses found in the B and F pockets, using both methimazole (MMZ) and propylthiouracil (PTU), for each of the alleles.


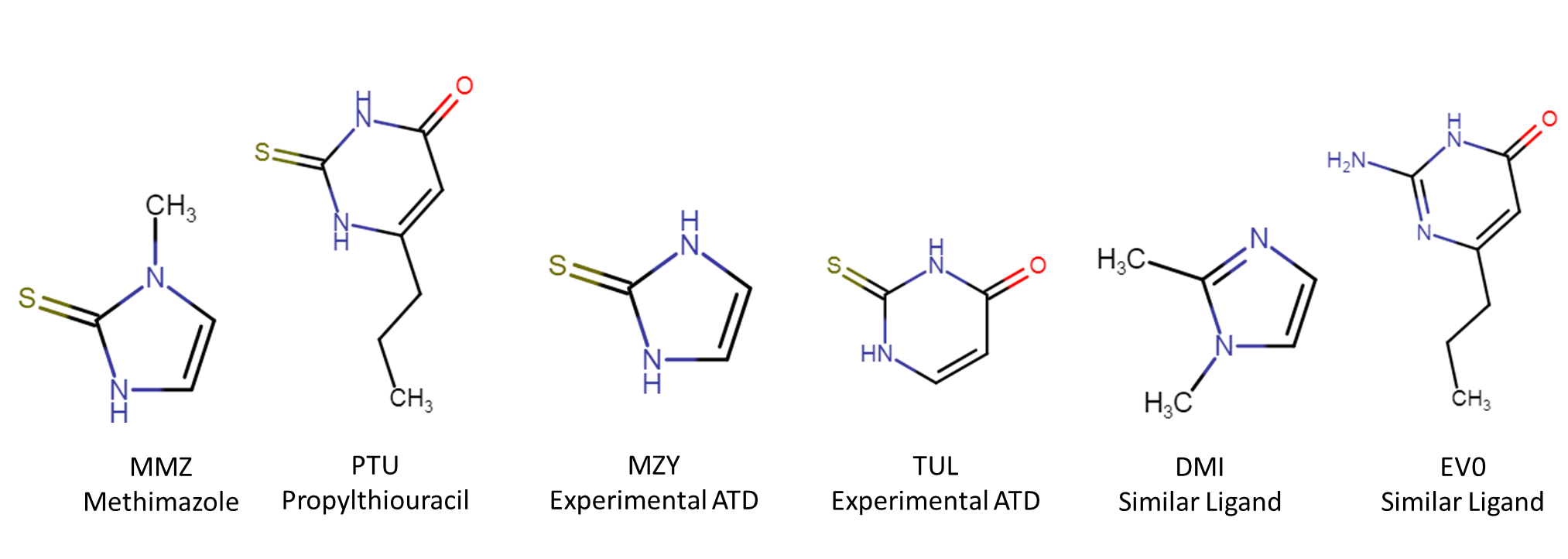


**Figure F: Structure of investigated drugs.** Structure of associated drugs methimazole and propylthiouracil along with similar ligands, some of which have been used as experimental anti-thyroid drugs.


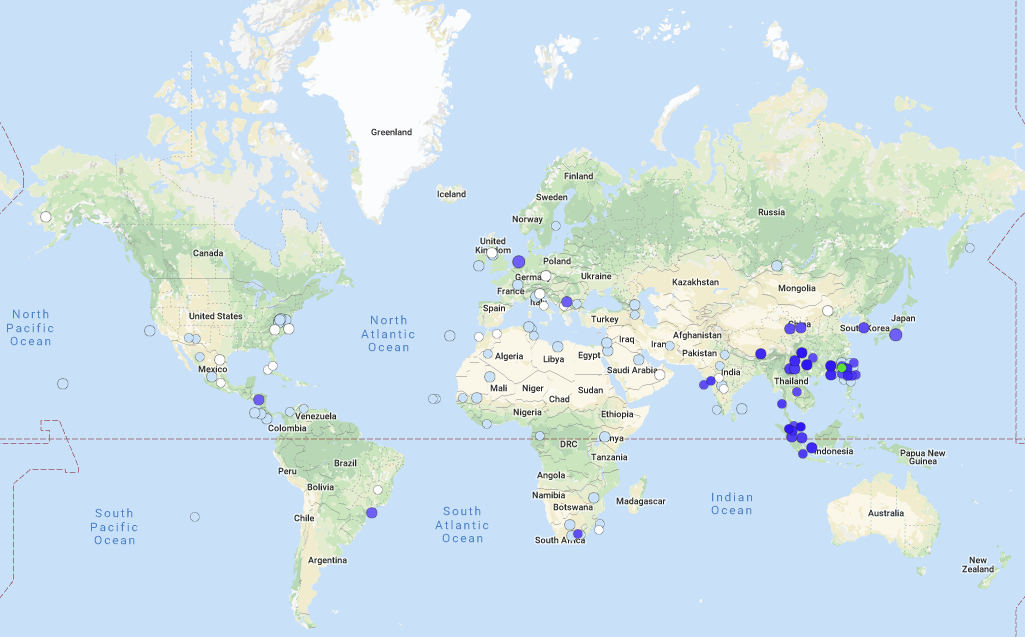

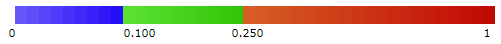


**Figure G: Allele frequency distribution for B*38:02.** Allele frequency distribution map obtained from AFND [4]. The size of the circles represent the sample size of each population with the colour representing the allele frequency, as shown by the key, with low frequency alleles shown in blue, mid in green and high in orange/red.


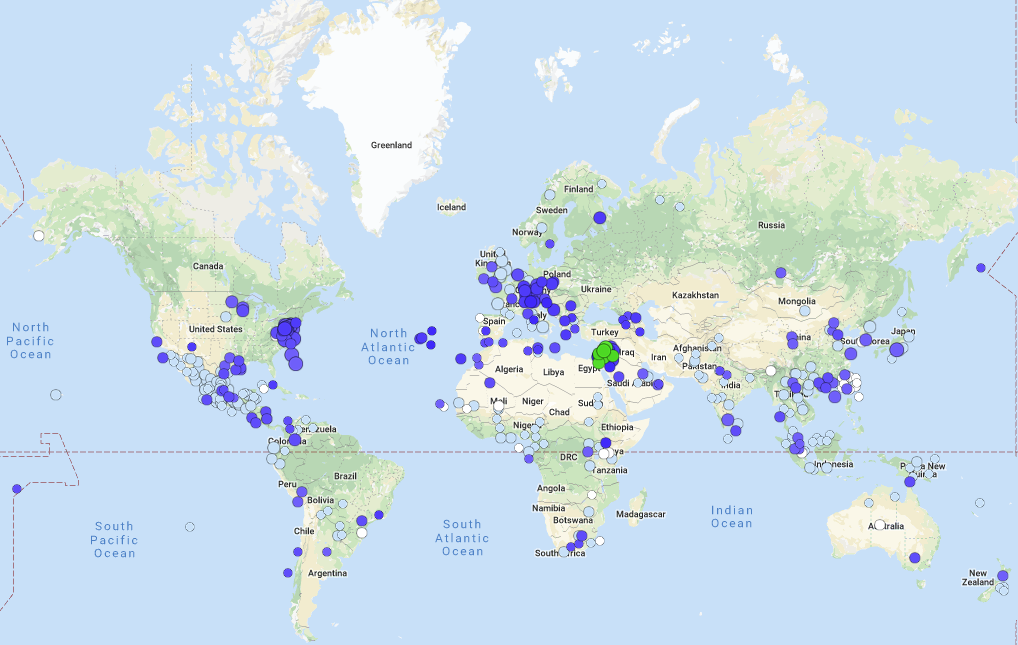

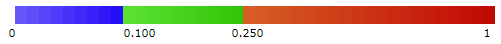


**Figure H: Allele frequency distribution for B*38:01.** Allele frequency distribution map obtained from AFND [4]. The size of the circles represent the sample size of each population with the colour representing the allele frequency, as shown by the key, with low frequency alleles shown in blue, mid in green and high in orange/red.


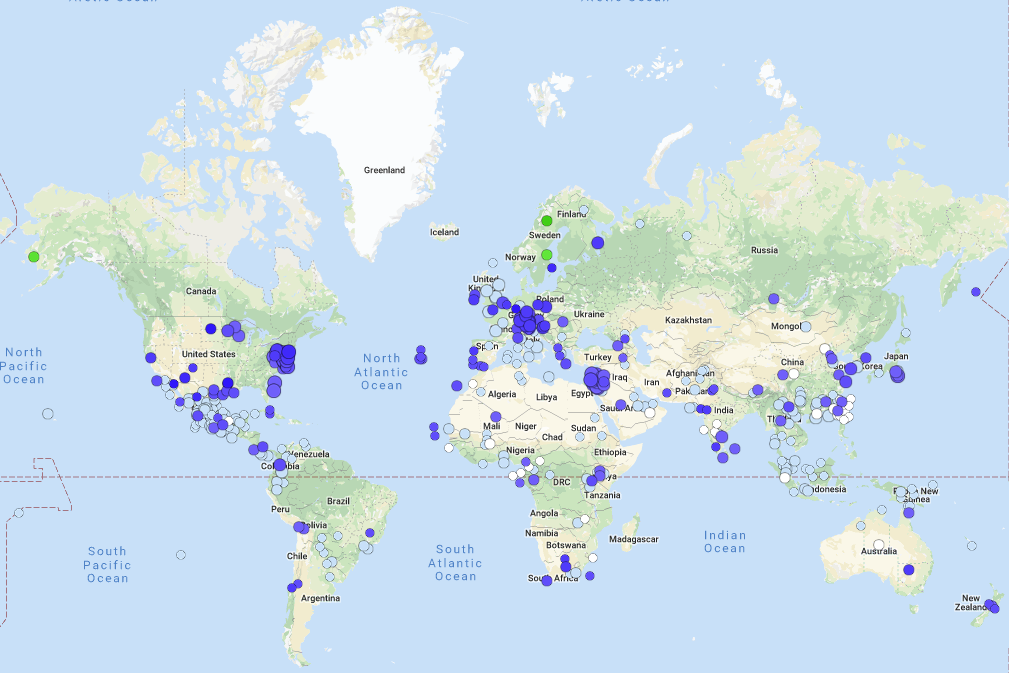

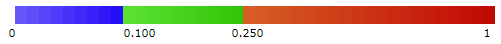


**Figure I: Allele frequency distribution for B*27:05.** Allele frequency distribution map obtained from AFND [4]. The size of the circles represent the sample size of each population with the colour representing the allele frequency, as shown by the key, with low frequency alleles shown in blue, mid in green and high in orange/red.

*
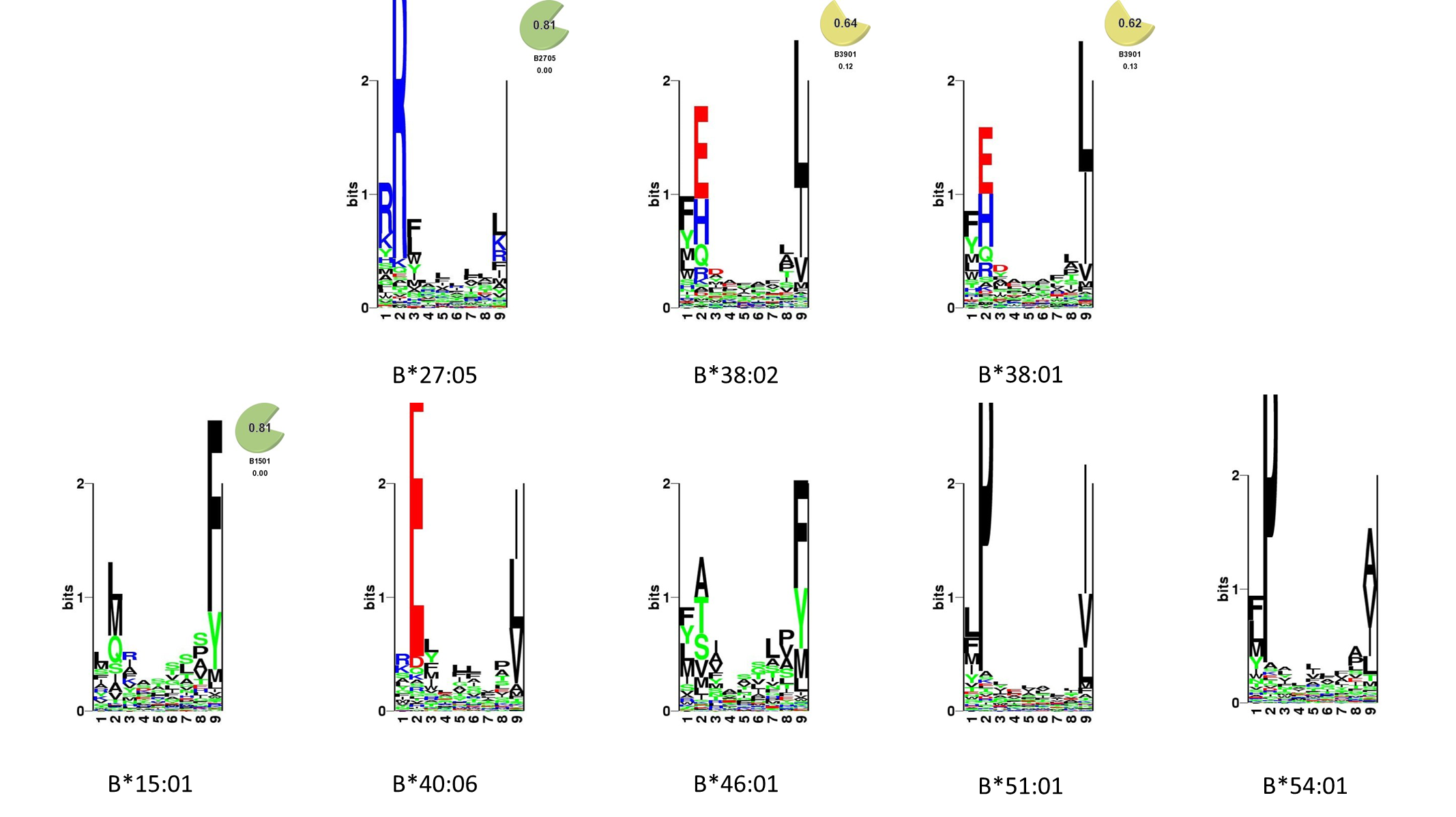
*

***Figure J: MHC motif viewer output for risk and control alleles.*** *MHC Motif Viewer outputs for B*27:05 and B*38:02 risk alleles and B*38:01 suspected risk alleles, showing predicted peptide binding motifs for each allele. Amino acids are coloured according to their physicochemical properties; acidic (D, E) coloured red, basic (H, K, R) coloured blue, hydrophobic (A, C, F, I, L, M, P, V, W) coloured black and Neutral (G, N, Q, S, T, Y) coloured green. The height of the column of letters is equal to the information content at that position and the height of the letter within the column is proportional to the frequency of the corresponding amino acid at that position. Where available, the reliability index is shown in the centre of the circle above the logo plot and is given as the estimated Pearson correlation coefficient for neural network predictions on the given alleles. The closest neighbour is also shown along with the distance to this neighbouring allele [22].*

*
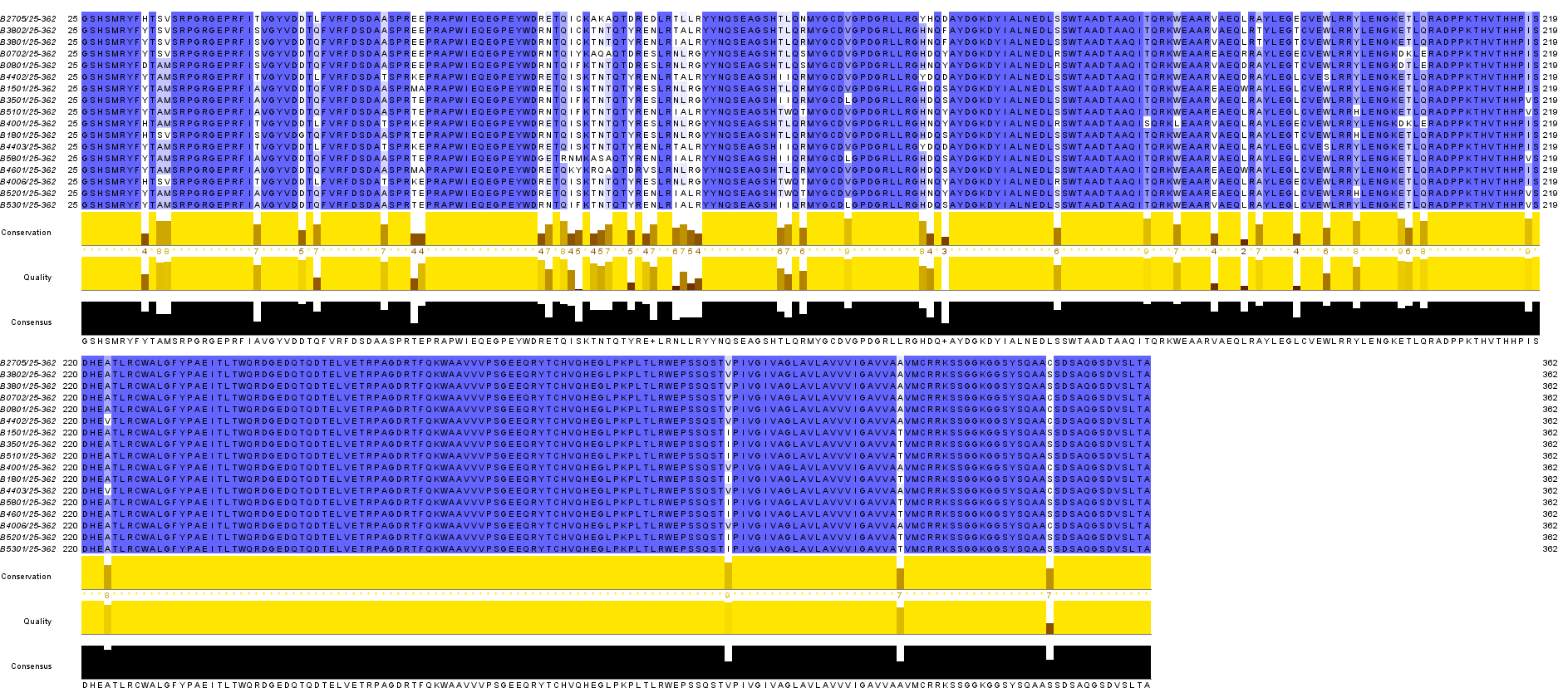

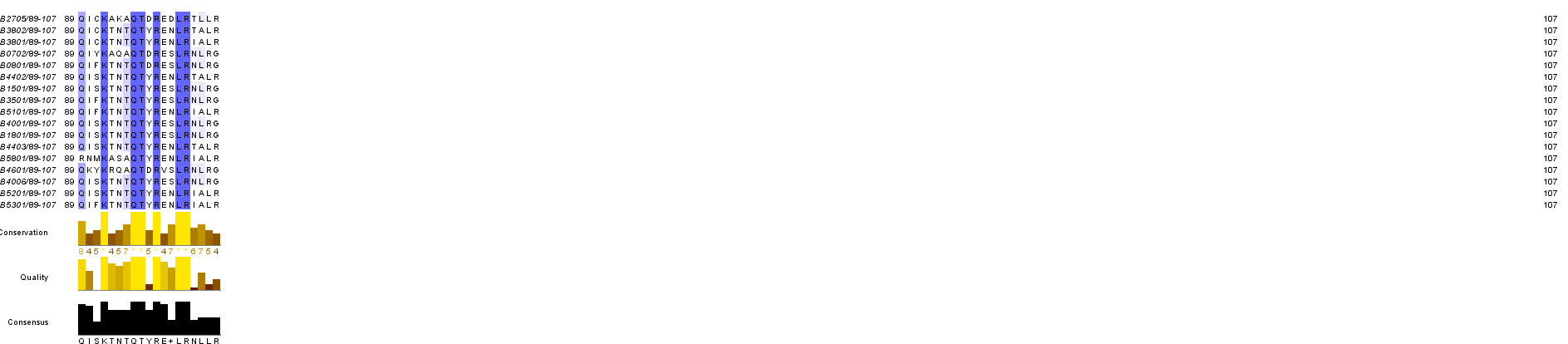
*

b)

a)

*
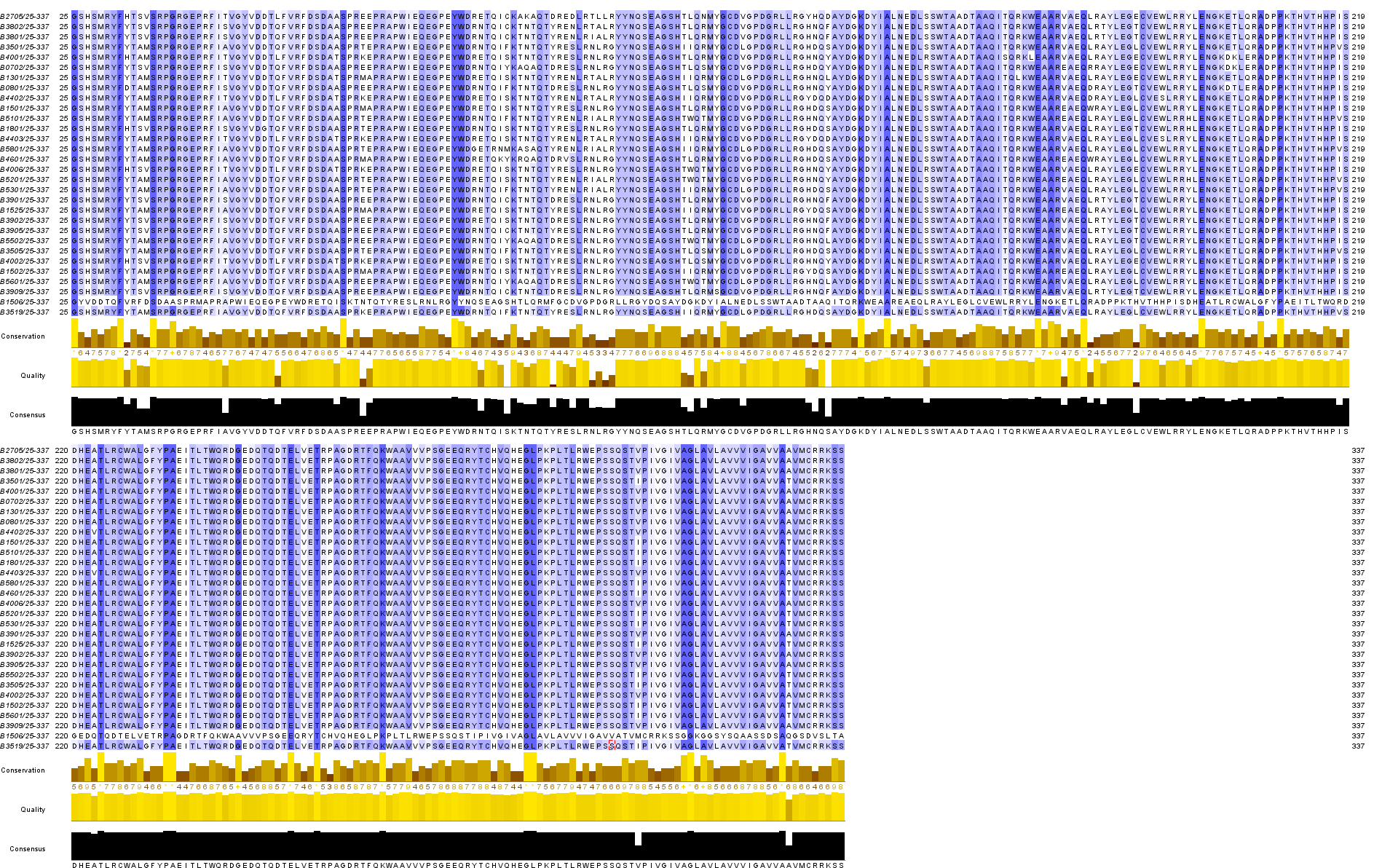

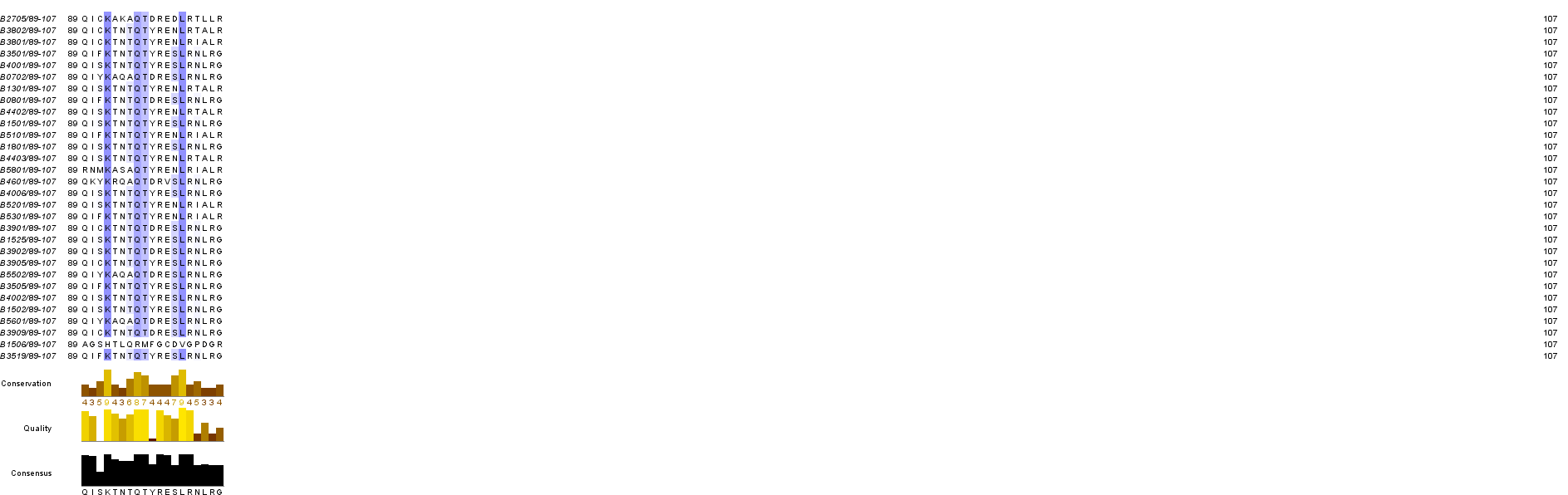
*

d)

c)

***Figure K: Multiple sequence alignment for risk, control and common alleles.*** *(a) Multiple sequence alignment for risk alleles B*27:05 and B*38:02, suspected risk allele B*38:01 and top 10 most frequent alleles for Caucasian, Asian populations selected from AFND and NMDP populations (repeats removed). (b) Focusing on positions 65-83. (c) Multiple sequence alignment for risk alleles B*27:05 and B*38:02, along with suspected risk allele B*38:01 and AFND top 20 most common alleles and NMDP top 10 most common for Caucasian, Asian and all populations (repeats removed – 30 unique sequences including risk alleles). (d) Focusing on positions 65-83. Positions 67 and 80 highlighted in red.*

*
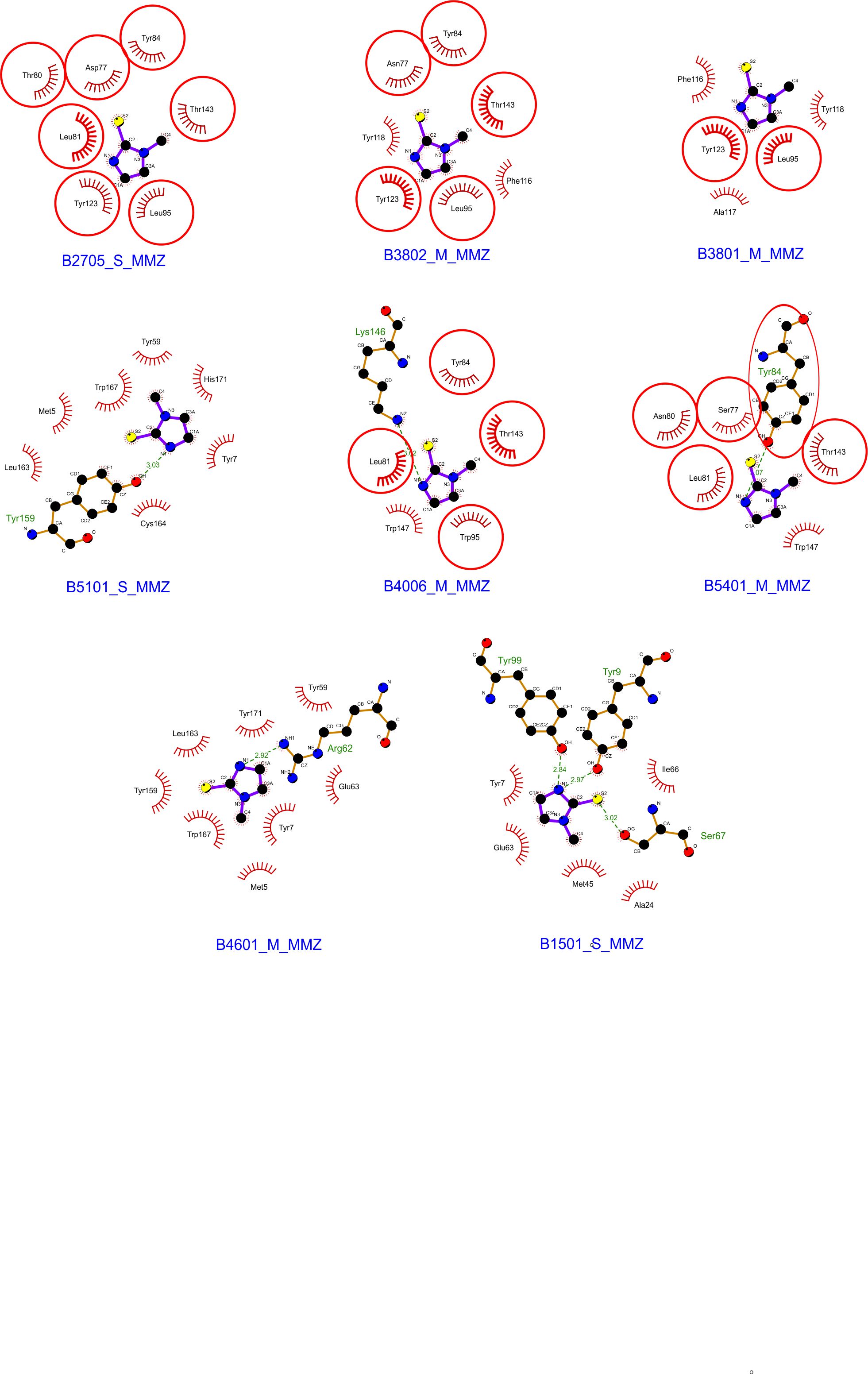
*

***Figure L: LigPlot figures for methimazole.*** *LigPlot figures show the interactions between methimazole and the residues on each allele for each of the risk alleles B*27:05 (N80T) and B*38:02 (N80T), suspected risk allele B*38:01 (N80I) and control alleles B*15:01 (N80), B*40:06 (N80), B*46:01 (N80), B*46:01 (N80), B*51:01 (N80I) and B*54:01 (N80), for the top scoring pose searching the peptide binding groove. Circles show comparisons between alleles, amino-acids at positions found interacting for B*27:05 highlighted with red circle.* *Hydrogen bonds are shown by green dotted lines.*

*
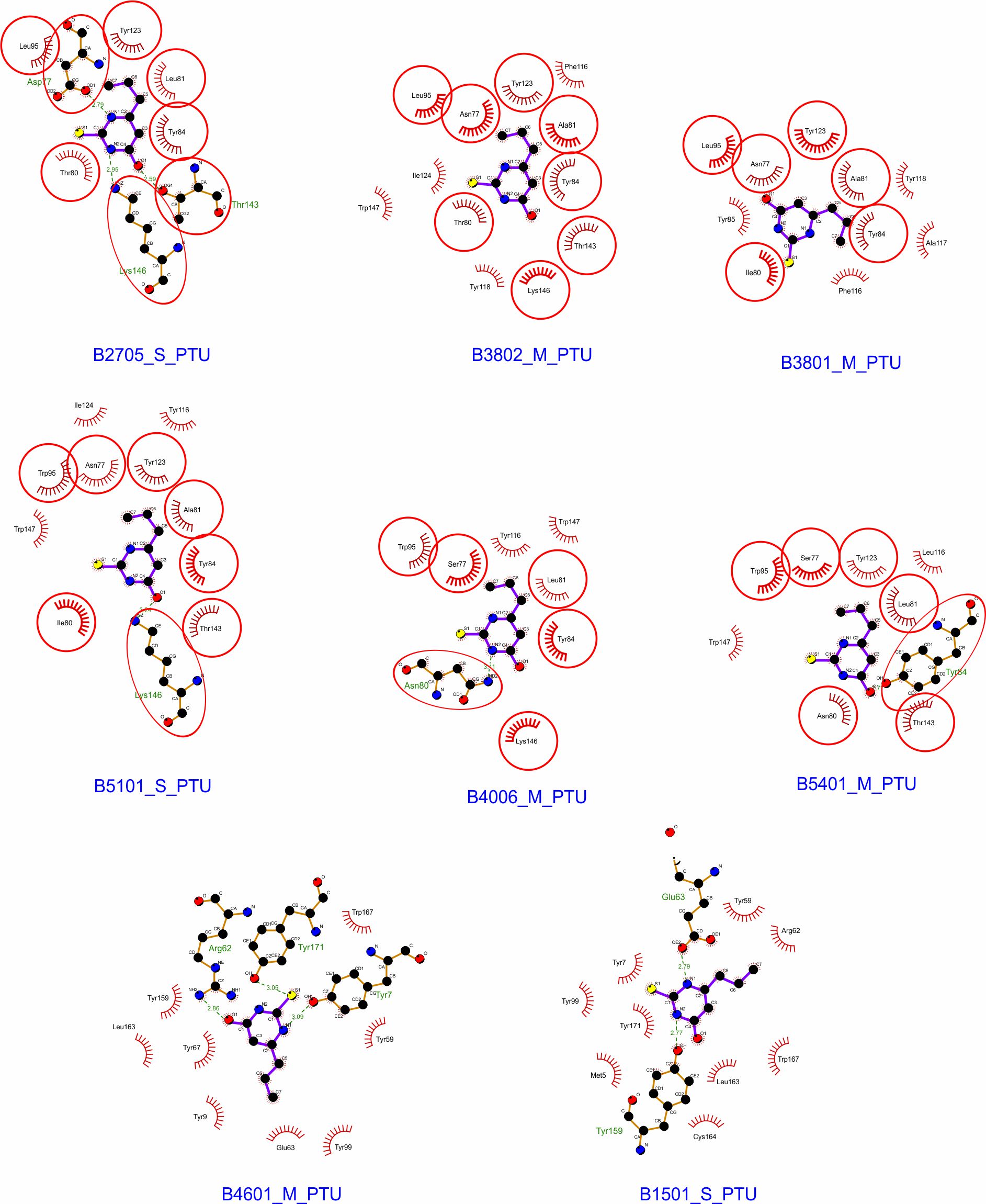
*

**Figure M: LigPlot figures for propylthiouracil.** LigPlot figures show the interactions between propylthiouracil and the residues on each allele for each of the risk alleles B*27:05 (N80T) and B*38:02 (N80T), suspected risk allele B*38:01 (N80I) and control alleles B*15:01 (N80), B*40:06 (N80), B*46:01 (N80), B*46:01 (N80), B*51:01 (N80I) and B*54:01 (N80), for the top scoring pose searching the peptide binding groove. Circles show comparisons between alleles, amino-acids at positions found interacting for B*27:05 highlighted with red circle. Hydrogen bonds are shown by green dotted lines.

*
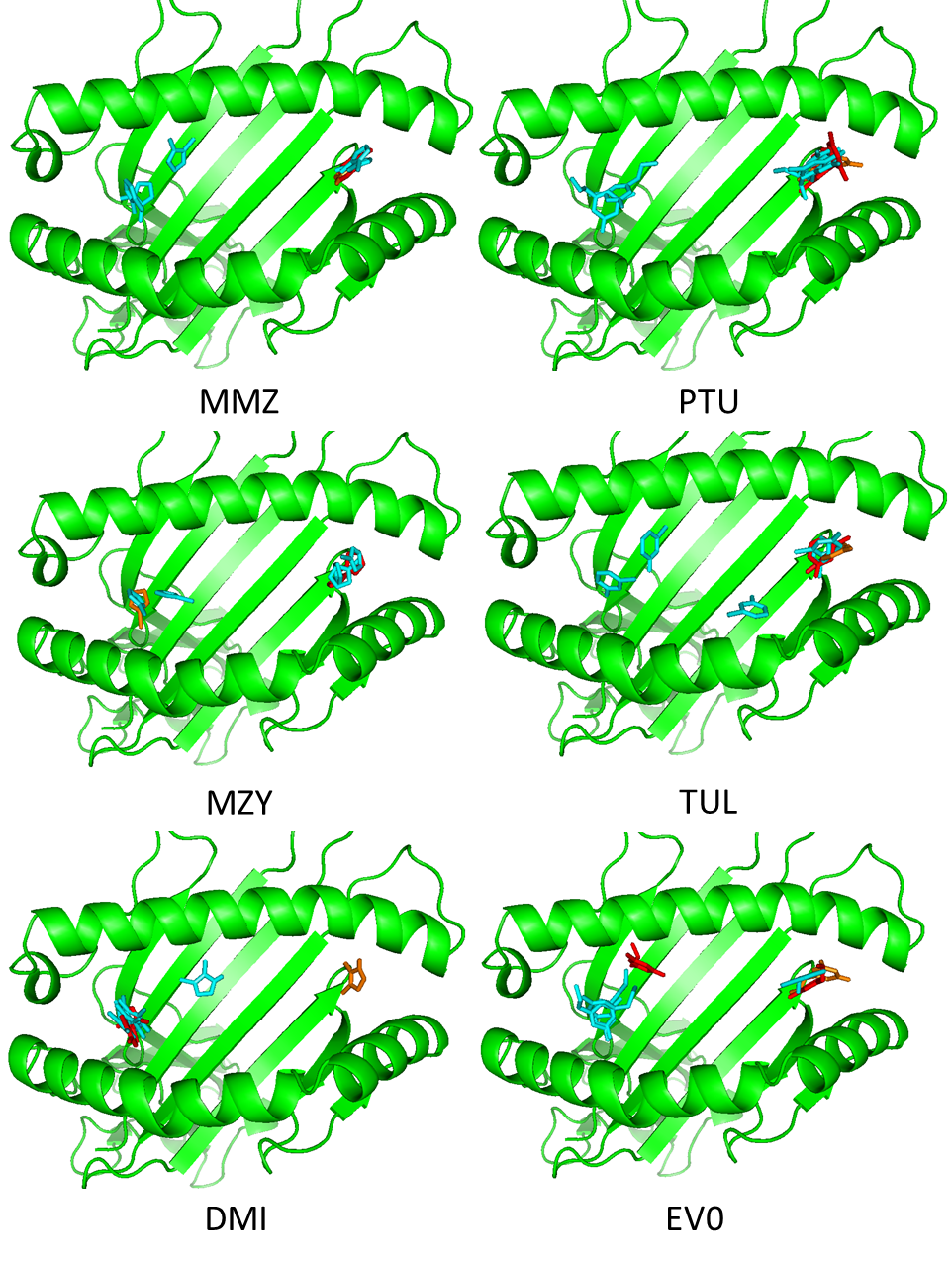
*

**Figure N: Predicted binding poses for investigated ligands.** Top scoring binding poses for B*27:05_S searching the peptide binding groove for each of the investigated drugs. Red poses show associated allele poses, orange shows B*38:01_M non-associated allele poses and blue the control allele poses.


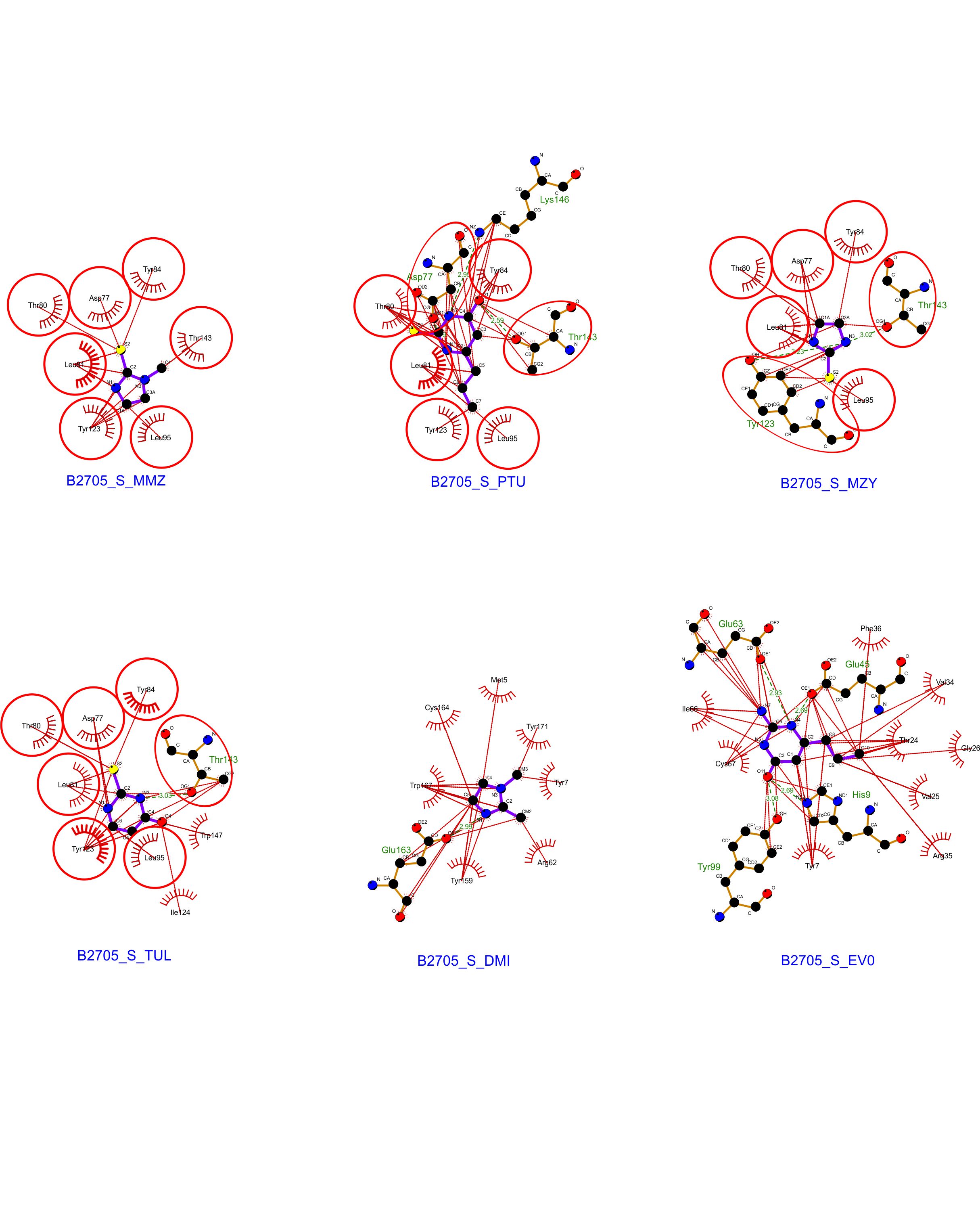


**Figure O: LigPlot figures for B*27:05.** LigPlot figures show the interactions between B*27:05 and each of the investigated ligands for the top scoring pose searching the peptide binding groove. Circles show comparisons between alleles, amino-acids at positions found interacting for MMZ highlighted with red circle. Hydrogen bonds shown by green dotted lines. Hydrophobic bonds shown in red.


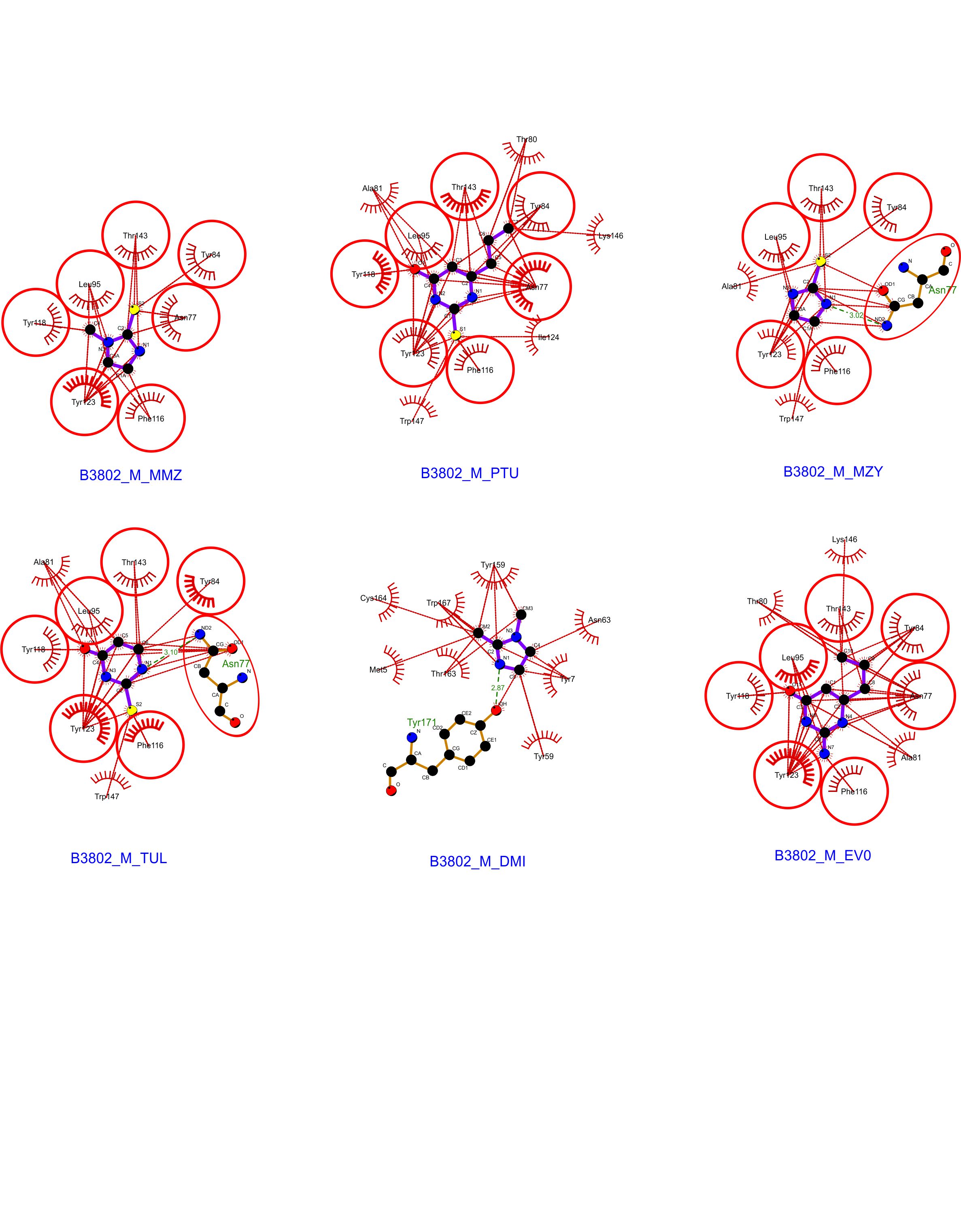


**Figure P: LigPlot figures for B*38:02.** LigPlot figures show the interactions between B*38:02 and each of the investigated ligands for the top scoring pose searching the peptide binding groove. Circles show comparisons between alleles, amino-acids at positions found interacting for MMZ highlighted with red circle. Hydrogen bonds shown by green dotted lines. Hydrophobic bonds shown in red.


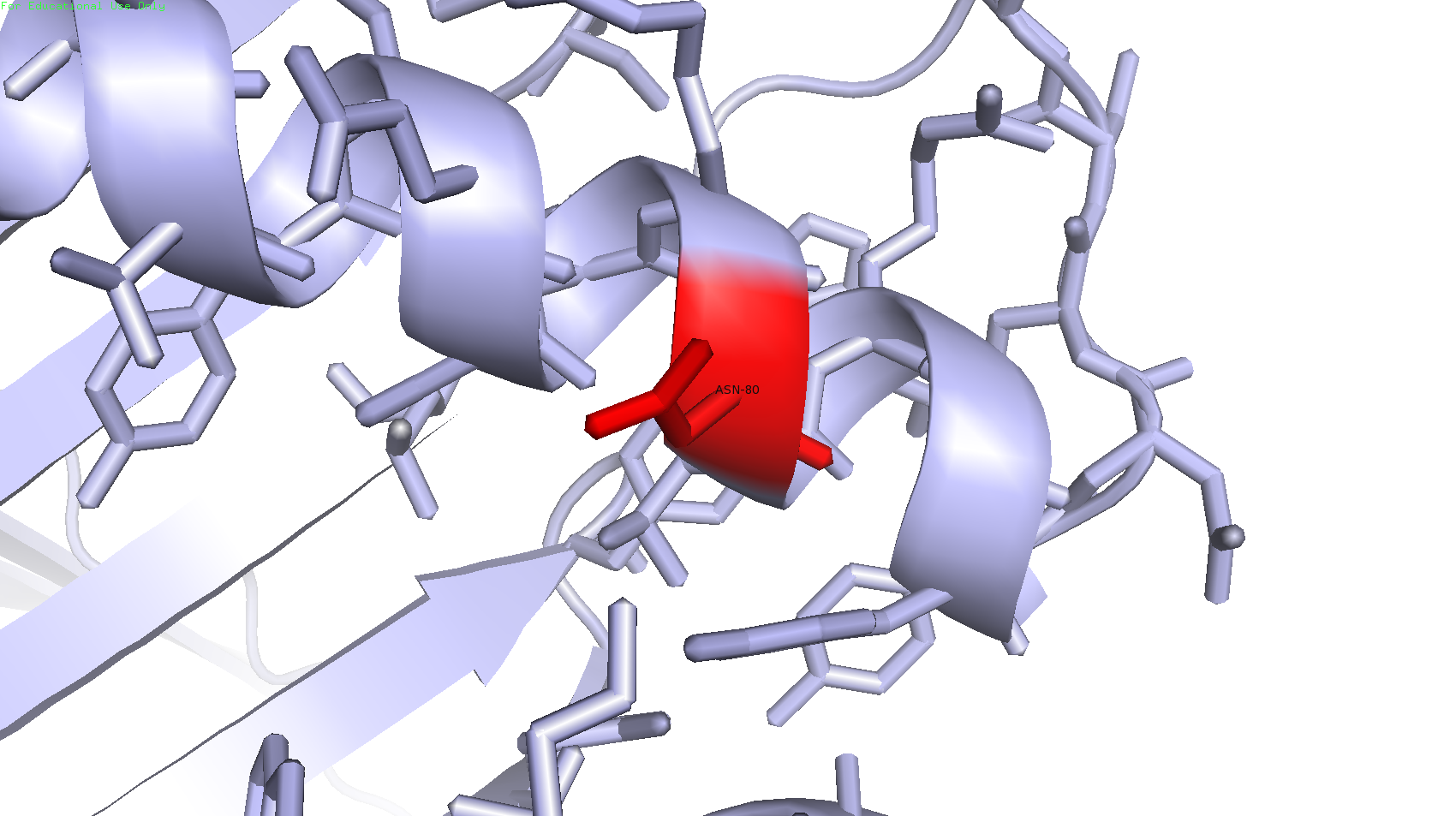

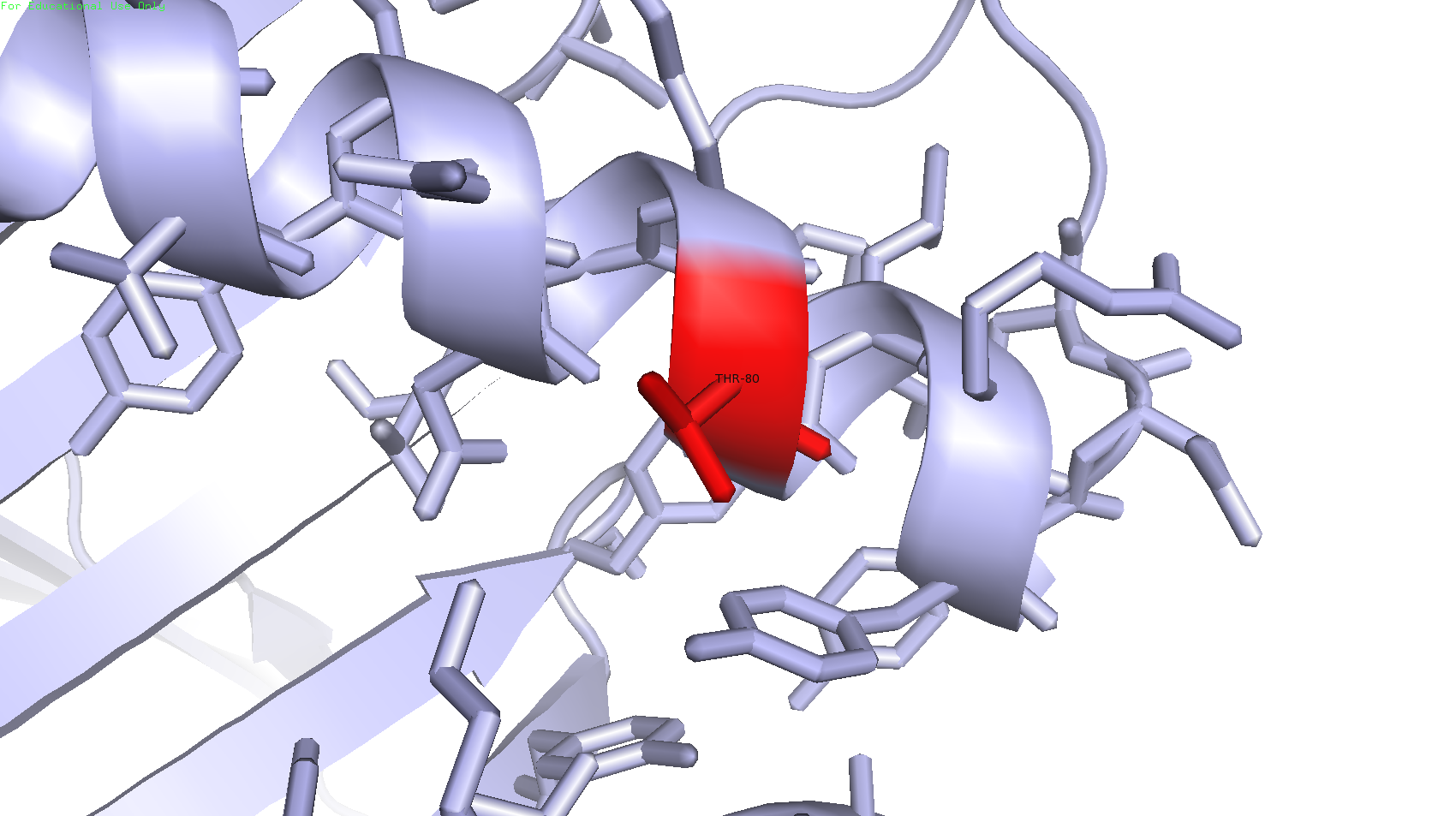


b)

a)

***Figure Q: Position 80 and neighbouring residues.*** *Position 80 (shown in red) and neighbouring F-pocket residues for a) B1501_S crystal structure and b) B3802_M modelled structure.*

1. Hallberg P, Eriksson N, Ibanez L, Bondon-Guitton E, Kreutz R, Carvajal A, et al. Genetic variants associated with antithyroid drug-induced agranulocytosis: a genome-wide association study in a European population. Lancet Diabetes Endocrinol. 2016;4(6):507-16. doi: 10.1016/S2213-8587(16)00113-3. PubMed PMID: 27157822.

2. Chen PL, Shih SR, Wang PW, Lin YC, Chu CC, Lin JH, et al. Genetic determinants of antithyroid drug-induced agranulocytosis by human leukocyte antigen genotyping and genome-wide association study. Nat Commun. 2015;6:7633. doi: 10.1038/ncomms8633. PubMed PMID: 26151496; PubMed Central PMCID: PMCPMC4506516.

3. He Y, Zheng J, Zhang Q, Hou P, Zhu F, Yang J, et al. Association of HLA-B and HLA-DRB1 polymorphisms with antithyroid drug-induced agranulocytosis in a Han population from northern China. Sci Rep. 2017;7(1):11950. doi: 10.1038/s41598-017-12350-2. PubMed PMID: 28931918; PubMed Central PMCID: PMCPMC5607267.

4. Gonzalez-Galarza FF, Takeshita LY, Santos EJ, Kempson F, Maia MH, da Silva AL, et al. Allele frequency net 2015 update: new features for HLA epitopes, KIR and disease and HLA adverse drug reaction associations. Nucleic Acids Res. 2015;43(Database issue):D784-8. doi: 10.1093/nar/gku1166. PubMed PMID: 25414323; PubMed Central PMCID: PMC4383964.

5. Sidney J, Peters B, Frahm N, Brander C, Sette A. HLA class I supertypes: a revised and updated classification. BMC Immunol. 2008;9:1. doi: 10.1186/1471-2172-9-1. PubMed PMID: 18211710; PubMed Central PMCID: PMCPMC2245908.

6. Saper MA, Bjorkman PJ, Wiley DC. Refined structure of the human histocompatibility antigen HLA-A2 at 2.6 A resolution. J Mol Biol. 1991;219(2):277-319. PubMed PMID: 2038058.

7. Hulsmeyer M, Fiorillo MT, Bettosini F, Sorrentino R, Saenger W, Ziegler A, et al. Dual, HLA-B27 subtype-dependent conformation of a self-peptide. J Exp Med. 2004;199(2):271-81. doi: 10.1084/jem.20031690. PubMed PMID: 14734527; PubMed Central PMCID: PMCPMC2211767.

8. Kumar P, Vahedi-Faridi A, Saenger W, Merino E, Lopez de Castro JA, Uchanska-Ziegler B, et al. Structural basis for T cell alloreactivity among three HLA-B14 and HLA-B27 antigens. J Biol Chem. 2009;284(43):29784-97. doi: 10.1074/jbc.M109.038497. PubMed PMID: 19617632; PubMed Central PMCID: PMCPMC2785609.

9. Sun M, Liu J, Qi J, Tefsen B, Shi Y, Yan J, et al. Nalpha-terminal acetylation for T cell recognition: molecular basis of MHC class I-restricted nalpha-acetylpeptide presentation. J Immunol. 2014;192(12):5509-19. doi: 10.4049/jimmunol.1400199. PubMed PMID: 24829406.

10. Rist MJ, Hibbert KM, Croft NP, Smith C, Neller MA, Burrows JM, et al. T Cell Cross-Reactivity between a Highly Immunogenic EBV Epitope and a Self-Peptide Naturally Presented by HLA-B*18:01+ Cells. J Immunol. 2015;194(10):4668-75. doi: 10.4049/jimmunol.1500233. PubMed PMID: 25855358.

11. Smith KJ, Reid SW, Harlos K, McMichael AJ, Stuart DI, Bell JI, et al. Bound water structure and polymorphic amino acids act together to allow the binding of different peptides to MHC class I HLA-B53. Immunity. 1996;4(3):215-28. PubMed PMID: 8624812.

12. Rist MJ, Theodossis A, Croft NP, Neller MA, Welland A, Chen Z, et al. HLA peptide length preferences control CD8+ T cell responses. J Immunol. 2013;191(2):561-71. doi: 10.4049/jimmunol.1300292. PubMed PMID: 23749632.

13. Toor JS, Rao AA, McShan AC, Yarmarkovich M, Nerli S, Yamaguchi K, et al. A Recurrent Mutation in Anaplastic Lymphoma Kinase with Distinct Neoepitope Conformations. Front Immunol. 2018;9:99. doi: 10.3389/fimmu.2018.00099. PubMed PMID: 29441070; PubMed Central PMCID: PMCPMC5797543.

14. Roder G, Blicher T, Justesen S, Johannesen B, Kristensen O, Kastrup J, et al. Crystal structures of two peptide-HLA-B*1501 complexes; structural characterization of the HLA-B62 supertype. Acta Crystallogr D Biol Crystallogr. 2006;62(Pt 11):1300-10. doi: 10.1107/S0907444906027636. PubMed PMID: 17057332.

15. Bade-Doding C, Theodossis A, Gras S, Kjer-Nielsen L, Eiz-Vesper B, Seltsam A, et al. The impact of human leukocyte antigen (HLA) micropolymorphism on ligand specificity within the HLA-B*41 allotypic family. Haematologica. 2011;96(1):110-8. doi: 10.3324/haematol.2010.030924. PubMed PMID: 20934997; PubMed Central PMCID: PMCPMC3012774.

16. Alpizar A, Marino F, Ramos-Fernandez A, Lombardia M, Jeko A, Pazos F, et al. A Molecular Basis for the Presentation of Phosphorylated Peptides by HLA-B Antigens. Mol Cell Proteomics. 2017;16(2):181-93. doi: 10.1074/mcp.M116.063800. PubMed PMID: 27920218; PubMed Central PMCID: PMCPMC5294207.

17. Chan KF, Gully BS, Gras S, Beringer DX, Kjer-Nielsen L, Cebon J, et al. Divergent T-cell receptor recognition modes of a HLA-I restricted extended tumour-associated peptide. Nat Commun. 2018;9(1):1026. doi: 10.1038/s41467-018-03321-w. PubMed PMID: 29531227; PubMed Central PMCID: PMCPMC5847591.

18. Tynan FE, Borg NA, Miles JJ, Beddoe T, El-Hassen D, Silins SL, et al. High resolution structures of highly bulged viral epitopes bound to major histocompatibility complex class I. Implications for T-cell receptor engagement and T-cell immunodominance. J Biol Chem. 2005;280(25):23900-9. doi: 10.1074/jbc.M503060200. PubMed PMID: 15849183.

19. Maenaka K, Maenaka T, Tomiyama H, Takiguchi M, Stuart DI, Jones EY. Nonstandard peptide binding revealed by crystal structures of HLA-B*5101 complexed with HIV immunodominant epitopes. J Immunol. 2000;165(6):3260-7. PubMed PMID: 10975842.

20. Menssen R, Orth P, Ziegler A, Saenger W. Decamer-like conformation of a nona-peptide bound to HLA-B*3501 due to non-standard positioning of the C terminus. J Mol Biol. 1999;285(2):645-53. doi: 10.1006/jmbi.1998.2363. PubMed PMID: 9878435.

21. Schrodinger, LLC. The PyMOL Molecular Graphics System, Version 1.8. 2015.

22. Rapin N, Hoof I, Lund O, Nielsen M. MHC motif viewer. Immunogenetics. 2008;60(12):759-65. doi: 10.1007/s00251-008-0330-2. PubMed PMID: 18766337; PubMed Central PMCID: PMCPMC2613509.
